# Postpandemic Cardiac Mortality Rates

**DOI:** 10.1001/jamanetworkopen.2025.12919

**Published:** 2025-05-30

**Authors:** Jason H. Wasfy, Yuqian Lin, Mary Price, Joseph P. Newhouse, Deborah Blacker, John Hsu

**Affiliations:** 1 Cardiology Division, Department of Medicine, Massachusetts General Hospital, Mass General Brigham, Harvard Medical School, Boston; 2 Mongan Institute for Population and Health Care Delivery Science, Department of Medicine, Massachusetts General Hospital, Mass General Brigham, Harvard Medical School, Boston; 3 Department of Health Care Policy, Harvard Medical School, Boston, Massachusetts; 4 Department of Health Policy and Management, Harvard T.H. Chan School of Public Health, Boston, Massachusetts; 5 Harvard Kennedy School, Cambridge, Massachusetts; 6 National Bureau of Economic Research, Cambridge, Massachusetts; 7 Department of Psychiatry, Massachusetts General Hospital, Mass General Brigham, Harvard Medical School, Boston; 8 Department of Epidemiology, Harvard T.H. Chan School of Public Health, Boston, Massachusetts

## Abstract

This cohort study describes the monthly and annual population-based cardiac mortality rates during and after the COVID-19 pandemic.

## Introduction

Studies using hospital data from 15 countries report a remarkably consistent 20% to 34% reduction in acute myocardial infarction (AMI) hospitalizations after the start of the COVID-19 pandemic.^1,2^ Cardiac procedures also decreased, while inpatient ST-segment elevation MI (STEMI) mortality increased in some countries.^3^ It is unclear whether these findings reflect actual changes in event rates, changes in hospital use, or limitations in using data restricted to hospitalized inpatients. To address these issues, we describe monthly and annual population-based cardiac mortality rates using state death certificate data and report actual and expected monthly cardiac death rates during and after the COVID-19 pandemic.

## Methods

This cohort study was approved by the Mass General Brigham Institutional Review Board and followed the Strengthening the Reporting of Observational Studies in Epidemiology (STROBE) reporting guideline. Consent was not required because of minimal risk.

We queried death certificate data for Massachusetts decedents (January 2014 to July 2024), which are stratified by location of death.^4^ We examined monthly cardiac deaths over the observation period. We then obtained total, age-specific and sex-specific mid-year Massachusetts population estimates from 2014 to 2023 from the US Census^5^ and used the age and sex distributions to standardize the population estimates with the 2014 Massachusetts population as reference. We then repeated the analyses stratified by the location of death.

To estimate expected monthly cardiac death rates, we fitted negative binomial regression models with data from 2014 to 2019, including age group, sex, month, year, and log of population size. We present the observed and expected age- and sex-standardized monthly cardiac deaths per 100 000 residents. We then repeated the analyses stratified by the location of death. Detailed methods appear in the eMethods in Supplement 1.

## Results

**Figure 1.**
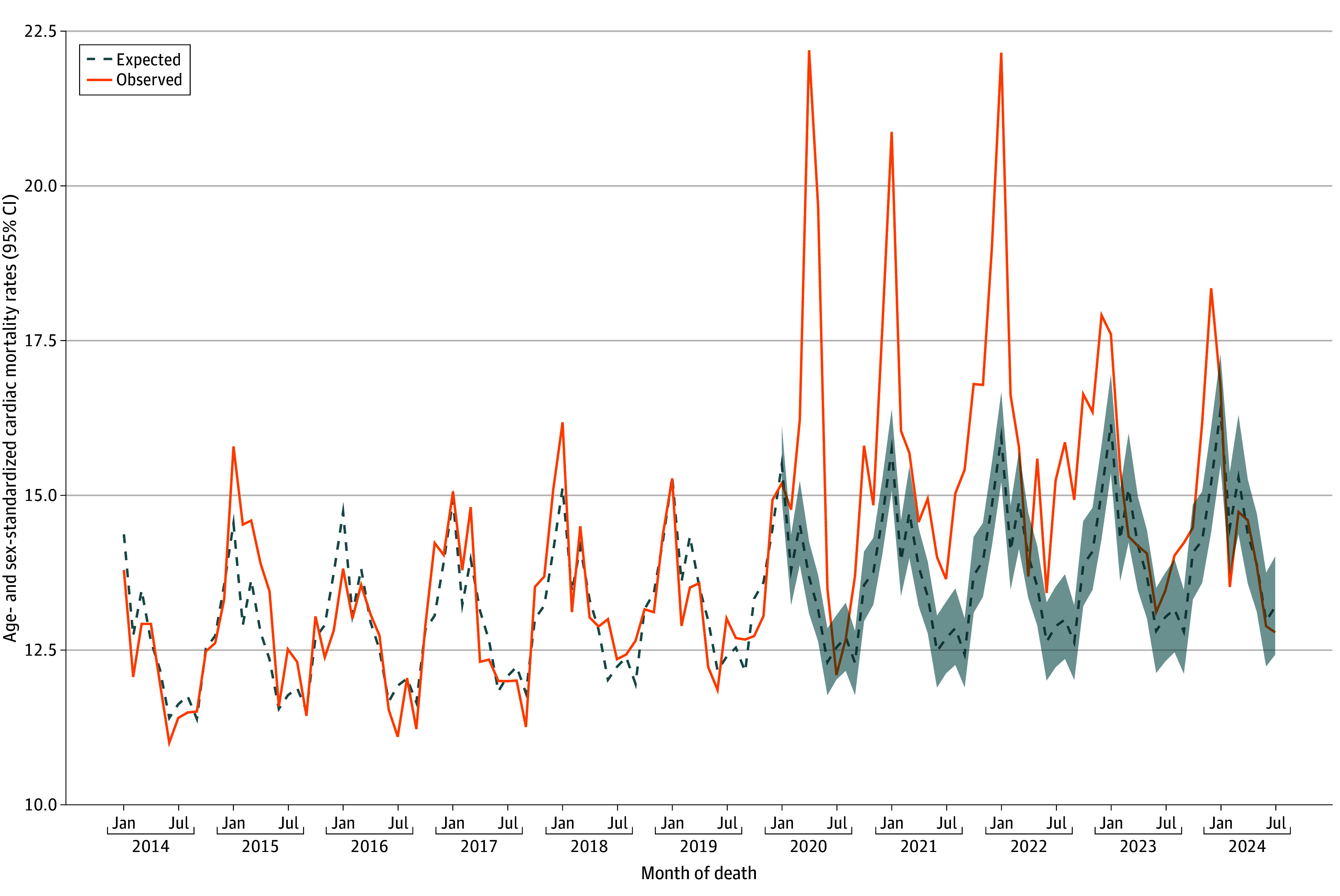
Age- and Sex-Standardized Cardiac Mortality Rates Per 100 000 Massachusetts Residents, January 2014 to July 2024 The solid orange line indicates the observed monthly age- and sex-standardized cardiac mortality rate, and the blue dashed line represents expected monthly age- and sex-standardized cardiac mortality rate. The blue shaded area (2020 to 2024) indicates the bootstrapped 95% CI for the expected rates.

**Figure 2.**
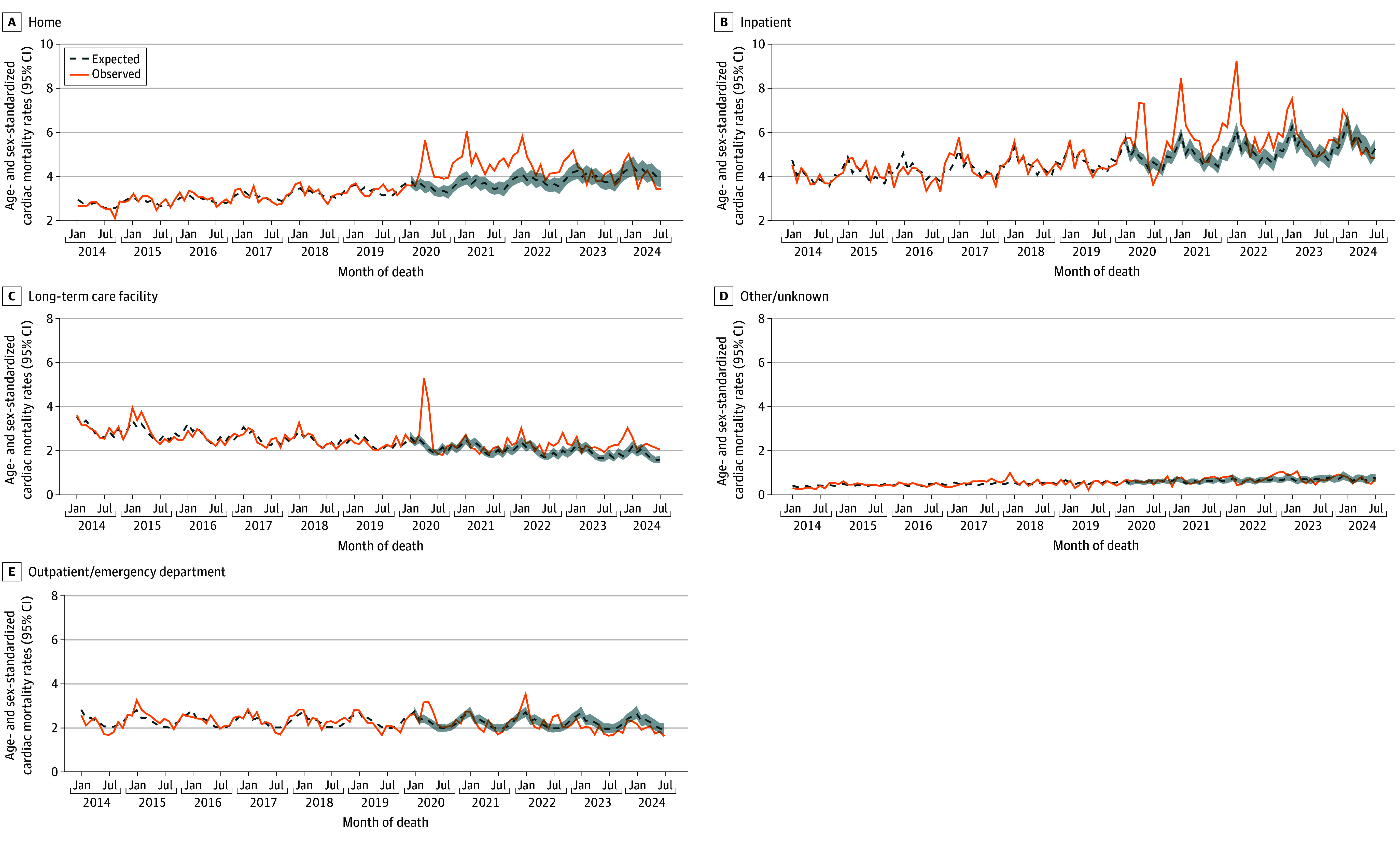
Observed and Expected Age- and Sex-Standardized Cardiac Mortality Rates Per 100 000 Massachusetts Residents, Stratified by Location of Death, January 2014 to July 2024 The solid orange line indicates the observed monthly age- and sex-standardized cardiac mortality rate for a given location, and the blue dashed line indicates the expected monthly age- and sex-standardized cardiac mortality rate for a given location. The blue shaded area (2020 to 2024) indicates the bootstrapped 95% CI for the expected rates.

Of 127 746 included individuals, mean (SD) age was 77 (14) years and 61 262 were female (47.9%). Figure 1 displays the observed and expected age- and sex-standardized total monthly cardiac mortality rates. Annual observed cardiac mortality exceeded expected from 2020 to 2023: 16% (95% CI, 13%-19%) higher in 2020, 17% (95% CI, 14%-21%) higher in 2021, 17% (95% CI, 13%-21%) higher in 2022, and 6% (95% CI, 2%-11%) higher in 2023. Stratified by location of death, monthly cardiac mortality rates exceeded expected for deaths at home between 2020 and 2022 and for deaths in hospitals between 2020 and 2023 (Figure 2).

## Discussion

In this population-based cohort study of Massachusetts decedents, we found cardiac deaths increased substantially starting in 2020, with exaggerated seasonal patterns and increases in deaths at home. While numerous other studies have found fewer admissions for cardiac emergencies in countries across the world, these studies may have missed events occurring outside of hospitals.

Taken together with prior studies, these results suggest increased cardiac mortality after the COVID-19 pandemic and changes in the locations of deaths. The UK has also reported sizeable increases in cardiac deaths at home in 2020.^6^ The US increase has persisted well past the early pandemic; as of mid-2024, some monthly rates remain elevated.

This study has limitations. We cannot exclude the possibility of misclassification in cause of death, particularly during the high stress periods of the initial outbreak. Nevertheless, given the considerable US Centers for Disease and Control and Prevention investments in standardizing coding and state requirements for evaluating deaths, death certificate data remain one of the most reliable and comprehensive data sources. Additionally, this study design cannot distinguish among causes of increased cardiac death (eg, effects of hospital and outpatient capacity limitations, hospital avoidance, or other potential causes).

Cardiac mortality has increased in Massachusetts and persisted for years after the onset of the COVID-19 pandemic, with shifts in the location of deaths. Further work is needed to improve the resilience of cardiac care during future pandemics. In addition, we should not base estimates of epidemiological trends on acute hospital data alone, since access problems or other issues may shift those events to homes.

## Notes

Supplement 1.
eMethods.

Supplement 2.
Data Sharing Statement
