# Supplementary material for: Postpandemic Cardiac Mortality Rates
Source: JAMA Netw Open. 2025 May 30;8(5):e2512919. doi: 10.1001/jamanetworkopen.2025.12919 (PMC12125641; doi:10.1001/jamanetworkopen.2025.12919)
Supplement: Supplement 1. — eMethods. [file jamanetwopen-e2512919-s001.pdf]

Supplemental Online Content

Wasfy JH, Lin Y, Price M, Newhouse JP, Blacker D, Hsu J. Post-pandemic elevated and persistent cardiac mortality rates. *JAMA Netw Open*. 2025. 8(5):e2512919. doi:10.1001/jamanetworkopen.2025.12919

eMethods.

This supplemental material has been provided by the authors to give readers additional information about their work.

© 2025 Wasfy JH et al. *JAMA Network Open*.

eMethods.

Study Population, Data Source, and Assessment of Missing Data

The primary data source for this analysis is provisional death certificate data in Massachusetts. The data contain cause and location of death (home, long term care facility including nursing homes and assisted living facilities, outpatient/emergency department, inpatient, other, or unknown). Massachusetts requires the medical examiner to investigate any sudden or unobserved death, e.g., at home.[5] The CDC has used the ICD-10 coding system for identifying the cause of death since 1999.[6,7]

We considered death occurrences for all residents, independent of age, from January 1, 2014, to July 31, 2024, with a total of 647,096 entries. We had no restrictions on age. After excluding 28 occurrences where the age was recorded as 999 or the sex was unknown, the dataset included 647,068 occurrences. We then examined missing data for other fields.

There is no missing data for "Date of Death," "Age", or "Sex". There is minimal missingness for "Place of Death" (0.0002%), "Cause of Death" and "Underlying Cause of Death" (0.0355% and 0.0349%, respectively). This missingness is slightly more pronounced in June and July of 2024 (although it remains below 3%).

ICD-10 Codes Used to Identify Cardiac Deaths

We then identified clinical syndromes consistent with acute coronary syndromes (ACS) or syndromes that could result from missed acute ACS treatment (e.g., cardiac arrest). We did not include potential longer-term complications of missed ACS (e.g., heart failure). Syndromes included in the analysis were: a) all types of acute MI (I21.0, I21.1, I21.2, I21.3, I21.4, I21.9); b) unstable angina (I20.0); and c) all types of cardiac arrest (I46.0, I46.1, I46.9). We considered deaths with these conditions as either the underlying cause or contributing conditions.

Estimates of Population Denominator

Population estimates were then combined with death counts to calculate death rates.To report mortality rates accounting for total population size over time, we needed to obtain estimates for total Massachusetts population over time. We obtained total, age- and sex- specific mid-year population estimates from 2014 to 2023 for Massachusetts residents from the US Census Bureau Population Estimates Program. Only mid-year population estimates are available at a state level, so monthly deaths were standardized with mid-year estimates for the Massachusetts population. For the 2024 estimate,

© 2025 Wasfy JH et al. *JAMA Network Open*.

which was unavailable at time of analysis, we used the compound annual growth rates (CAGR) between 2021 and 2023, and project based on 2021 population estimates, adjusting for age and sex. Specifically, we calculated the CAGR between 2021 and 2023 for each age and sex group as follows:

$$\mathrm{CAGR}_{\mathrm{age,sex,2021-2023}} = \left(\frac{\mathrm{Population}_{\mathrm{age,sex,2023}}}{\mathrm{Population}_{\mathrm{age,sex,2021}}}\right)^{\frac{1}{2}} - 1$$

Using this CAGR, the 2024 population estimates for each age and sex group were derived as:

$$\mathrm{Population}_{\mathrm{age,sex,2024}} = \mathrm{Population}_{\mathrm{age,sex,2021}} \times \left(1 + \mathrm{CAGR}_{\mathrm{age,sex,2021-2023}}\right)^{3}$$

The total population estimate for Massachusetts in 2024 was then obtained by summing across all age and sex groups:

$$\mathrm{Population}_{\mathrm{total,2024}} = \sum_{\mathrm{age,sex}} \mathrm{Population}_{\mathrm{age,sex,2024}}$$

Construction of a Counterfactual Trend Post January 2020 from 2014-2019 Data

To estimate the expected number of monthly cardiac deaths in January 2020 through July 2024, we used predictions from a negative binomial regression model estimated using pre-period data (January 2014 to December 2019) and adjusting for age, sex and seasonality. We chose the negative binomial regression models due to the observed overdispersion in most groups. We estimated the coefficients and dispersion parameter using maximum likelihood estimation, implemented through the glm.nb function in the MASS package in R (RStudio 2022.07.2+576 'Spotted Wakerobin' Release, 2022-09-06, for Windows).

To account for seasonality in the data, we tested four different model specifications and selected the one with the lowest Akaike Information Criterion (AIC).

We specified the final model with month as a categorical variable and no winter term, yielding the lowest AIC. The negative binomial regression model for cardiac death counts per month follows this equation:

© 2025 Wasfy JH et al. *JAMA Network Open*.

$$\log(\mu_{m,a,s}) = \beta_0 + \sum_{a=1}^{5} \beta_{1,a}(\text{age})_a + \beta_2(\text{sex})_s + \beta_3(\text{year}) + \sum_{m=1}^{11} \beta_{4,m}\,(\text{month})_m$$
$$+ \log(\text{population size}_{y,a,s})$$

Where:

- $\mu_{m,a,s}$ is the expected cardiac death count for month m, age group a, and sex s.
- $\sum_{a=1}^{5} \beta_{1,a}$ represents the coefficients for each of the five age groups (excluding the reference group), and $(\text{age})_a$ is treated as categorical variables with six levels in total: <45 (reference), 45-54, 55-64, 65-74, 75-84, and ≥85.
- β_3 captures the annual trend for the cardiac death counts.
- $\sum_{m=1}^{11} \beta_{4,m}$ captures the seasonality of the cardiac death count, where $(\text{month})_m$ is treated as a categorical variable.
- $\log(\text{population size}_{y,a,s})$ is included as an offset (i.e., coefficient constrained to 1) to adjust for different population sizes of each age-sex group within each year y, remaining constant across months within the same year for each age-sex group.

To calculate the expected monthly age- and sex-standardized cardiac mortality rates (R_m^*) from January 2020 to July 2024, we followed a three-step process. **First,** we estimated the **expected cardiac death counts ($\mu_{m,a,s}$)** for each age-sex group (a, s) in each month (m) using the pre-specified negative binomial model fitted to the full post-period data. **Second,** we computed the **age- and sex- specific mortality rates ($R_{m,a,s}$)** by dividing the expected cardiac death counts ($\mu_{m,a,s}$) by the corresponding age- and sex- specific population estimates ($N_{y,a,s}$) :

$$R_{y,a,s} = \frac{\mu_{m,a,s}}{N_{y,a,s}}$$

where $N_{y,a,s}$ represents the mid-year population estimate for year y , age group a and sex s. Details on the data sources and adjustments for the 2024 population estimates were described in an earlier section. **Third**, we obtained the expected **age- and sex-standardized mortality rates (R_m^*)** by weighting the age- and sex-specific mortality rates ($R_{m,a,s}$) by the 2014 population structure. The formula for R_m^* is expressed as:

$$R_m^* = \sum_s \sum_a (R_{m,a,s} \times w_{a,s})$$

where $w_{a,s}$ is the proportion of the 2014 population in age group a and sex s. This weighting adjusts for the changes in the population age overtime and thus ensure comparability over time.

© 2025 Wasfy JH et al. *JAMA Network Open.*

In addition to the expected rates, we computed observed age- and sex-standardized mortality rates following the same standardization approach. Using the observed and expected standardized mortality rates, we calculated the ratio of observed to expected rates (O/E ratio) and the difference between observed and expected rates (O/E difference) for each month.

To construct the 95% confidence intervals (CIs) for the expected standardized mortality rates, O/E ratio, and O/E difference, we used a bootstrap approach with 2000 iterations. For each iteration, we repeated the same three-step process described above, except that in the first step, we estimated the expected age-sex specific death counts ($\mu_{m,a,s}$) from the negative binomial models fitted to the bootstrapped samples. We then used the resulting bootstrap distributions of the expected standardized mortality rates (R_m^*), OE ratios, and O/E differences to calculate the 2.5th and 97.5th percentiles as the lower and upper bounds of the 95% confidence intervals.

Similarly, we computed yearly estimates for expected standardized mortality rates, O/E ratios, and O/E differences. For point estimates, we added the annual age- and sex-specific death counts across all months and divided by the corresponding age- and sex-specific population estimates to obtain the yearly age- and sex- specific death rates. Since the denominator was the same for every month of the year (given the assumption that mid-year population estimate applied to every month of the year) the monthly death rates could be added to produce a yearly estimate.

$$R_{y,a,s} = \frac{\sum_m \mu_{m,a,s}}{N_{y,a,s}}$$

Subsequently, we standardized these yearly expected mortality rates ($R_{y,a,s}$) to 2014 population structure at the age and sex level.

$$R_y^* = \sum_s \sum_a (R_{y,a,s} \times w_{y,s})$$

We obtained confidence intervals for the yearly expected standardized mortality rates, O/E rate ratios, and O/E rate differences using the 2.5th and 97.5th percentiles of the corresponding bootstrap distributions derived from 2000 iterations.

Sensitivity Analyses

Binary exposure variable

We conducted a sensitivity analysis with a binary exposure variable. In this new analysis, we define pre-COVID as January 2014-December 2019, COVID and post-COVID as Jan 2020-July 2024.

Binary Exposure Variable and Interaction with Location Variables

We also conducted a sensitivity analysis including the binary variable discussed above and using interaction variables with location of death.

© 2025 Wasfy JH et al. *JAMA Network Open*.
