# Supplementary material for: Postpandemic Cardiac Mortality Rates
Source: JAMA Netw Open. 2025 May 30;8(5):e2512919. doi: 10.1001/jamanetworkopen.2025.12919 (PMC12125641; doi:10.1001/jamanetworkopen.2025.12919)
Supplement: Supplement 2. — Data Sharing Statement [file jamanetwopen-e2512919-s002.pdf]

Data Sharing Statement

Wasfy. Postpandemic Cardiac Mortality Rates. *JAMA Netw Open*. Published May 30, 2025.
doi:10.1001/jamanetworkopen.2025.12919

Data
Data available: No

Additional Information
Explanation for why data not available: Not allowed per data use agreement.
